# Modification of sperm morphology during long-term sperm storage in the reproductive tract of the Chinese soft-shelled turtle, *Pelodiscus sinensis*

**DOI:** 10.1038/srep16096

**Published:** 2015-11-05

**Authors:** Linli Zhang, Ping Yang, Xunguang Bian, Qian Zhang, Shakeeb Ullah, Yasir Waqas, Xiaowu Chen, Yi Liu, Wei Chen, Yuan Le, Bing Chen, Shuai Wang, Qiusheng Chen

**Affiliations:** 1 Laboratory of Animal Cell Biology and Embryology, College of Veterinary Medicine, Nanjing Agricultural University, Nanjing, Jiangsu 210095, PR China

## Abstract

Sperm storage *in vivo* extends the time window for fertilisation in several animal species, from a few days to several years. The underlying storage mechanisms, however, are largely unknown. In this study, spermatozoa from the epididymis and oviduct of Chinese soft-shelled turtles were investigated to identify potentially relevant morphological features and transformations at different stages of sperm storage. Large cytoplasmic droplets (CDs) containing lipid droplets (LDs) were attached to the midpiece of most spermatozoa in the epididymis, without migrating down the sperm tail. However, they were absent from the oviductal spermatozoa, suggesting that CDs with LDs may be a source of endogenous energy for epididymal spermatozoa. The onion-like mitochondria recovered their double-membrane morphology, with typical cristae, within the oviduct at a later stage of storage, thus implying that mitochondrial metabolism undergoes alterations during storage. Furthermore, a well developed fibrous sheath on the long principal piece was the integrating ultrastructure for glycolytic enzymes and substrates. These novel morphological characteristics may allow turtle spermatozoa to use diverse energy metabolism pathways at different stages of storage.

Sperm storage is a reproductive strategy widely used by animal species that extends the longevity of spermatozoa until the opportunity for fertilisation occurs. This in turn increases the likelihood of successful fertilization, particularly in some insect, fish, amphibian and reptile that store spermatozoa *in vivo* within the female reproductive tract for long periods (up to months and years)^1^,^2^,^3^. With the storage of spermatozoa, females may gain fitness benefits from multiple paternities over the long term. They may also be able to control the paternity of their offspring through the selective use of spermatozoa^4^. Male reproductive success is largely an effect of cryptic female choice during sperm storage in these species. Thus, spermatozoa stored in the female reproductive tract can outcompete more recently inseminated spermatozoa in subsequent ovarian cycles. In some reptiles, sperm storage affects sex allocation of offspring, and stored spermatozoa have a 23% higher probability of producing sons than daughters in some species of lizard^5^. In males, sperm maturation and storage within the epididymis differs among vertebrates due to a variation in the duration of spermatogenesis^6^. Spermatozoa are stored in the epididymis for few hours in birds to many months in reptiles and bats^7^.

With so many examples, the mechanisms involved in these disparate species should be relatively easy to identify, but that has not been the case^8^. The most detailed investigations of sperm storage mechanisms in the female reproductive tract have been performed in mammalian systems, in which sperm storage times are limited^9^. If we could determine the mechanisms behind these processes and thereby facilitate the long-term storage of spermatozoa at ambient temperatures, the practical benefits would be enormous.

Many of the species that store spermatozoa are threatened and therefore protected by law^3^. However, the Chinese soft-shelled turtle, *Pelodiscus sinensis*, is an abundant freshwater aquaculture reptile in China that is suitable as a model organism. Spermatogenesis in this turtle is seasonal, with spermiation occurring in October (late autumn in Nanjing)^10^. Next, immature spermatozoa are transferred into the epididymis, where they are stored until the following May. After mating activity and ovulation between June and August, the spermatozoa are stored in the oviduct until used to fertilise eggs the following year.

Reptilian species store spermatozoa in both female and male reproductive tracts^11^,^12^. Some female reptiles possess specialised sperm storage tubules (SSTs)^11^,^13^,^14^. In a previous study by our group, a similar storage structure was identified in the Chinese soft-shelled turtle^15^. The epithelial cells in the oviduct and the epididymis have distinctive secretive functions, which may contribute to the nourishment and protection of spermatozoa^15^,^16^. Although the storage environment is an important factor of sperm survival, another factor may be the gametes themselves. However, no exhaustive research on the individual spermatozoon during long-term storage has been conducted in the turtle.

The ability of spermatozoa to survive in the storage organs may influence fertilization success, so that optimal sperm morphology may maximize sperm longevity^17^. Alternately, the ultrastructure and swimming velocity of spermatozoa can influence sperm competition^18^. In insects, sexual selection via sperm competition favors increasing sperm length, with evolutionary consequences^1^. In addition, sperm morphology may indicate the status of physiological function. For instance, the CDs of spermatozoa cytoplasmic droplets (CDs), which contain a mixture of different enzymes^19^, may play a role in the acquisition of sperm motility during epididymal maturation^20^. The fibrous sheath functions as a scaffold for localized proteins involved in signalling cascades^21^,^22^, which are critical for normal flagellar function. The relationships among sperm storage, competition, morphology and physiology are extremely complex. More data on the behavioural and physiological aspects of spermatozoa form and function are required.

We hypothesized that, by studying the structural characteristics of spermatozoa at various stages of maturation and during long-term storage in both female and male, we would be able to use the physiological context to help interpret the results in terms of the mechanisms that permit long-term sperm storage. The Chinese soft-shelled turtle is particularly useful for this purpose because of the dramatic and measurable changes in sperm structure that occur during its storage.

## Results

**Figure 1:**
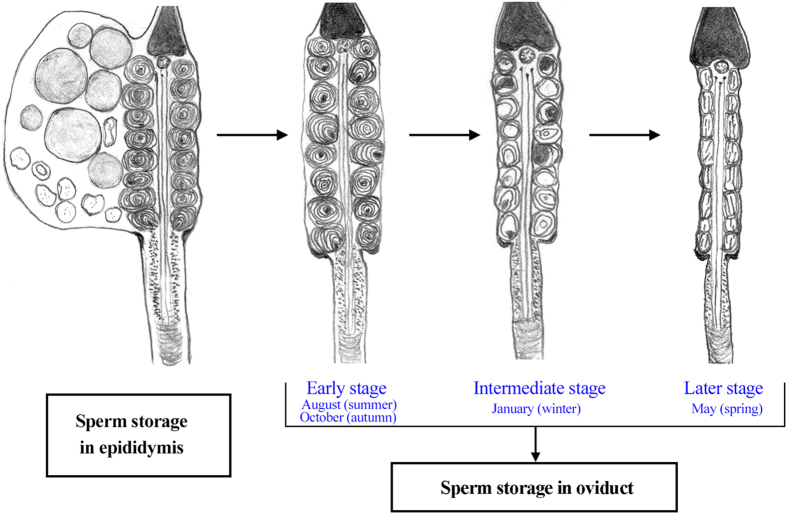
A schematic organization demonstrating the transformation of spermatozoa in the genital tract of the Chinese soft-shelled turtle. The CD with LDs and the cytoplasmic ring of the principal piece were lost and mitochondria were modified when transferred from the epididymis to the oviduct.

**Figure 2:**
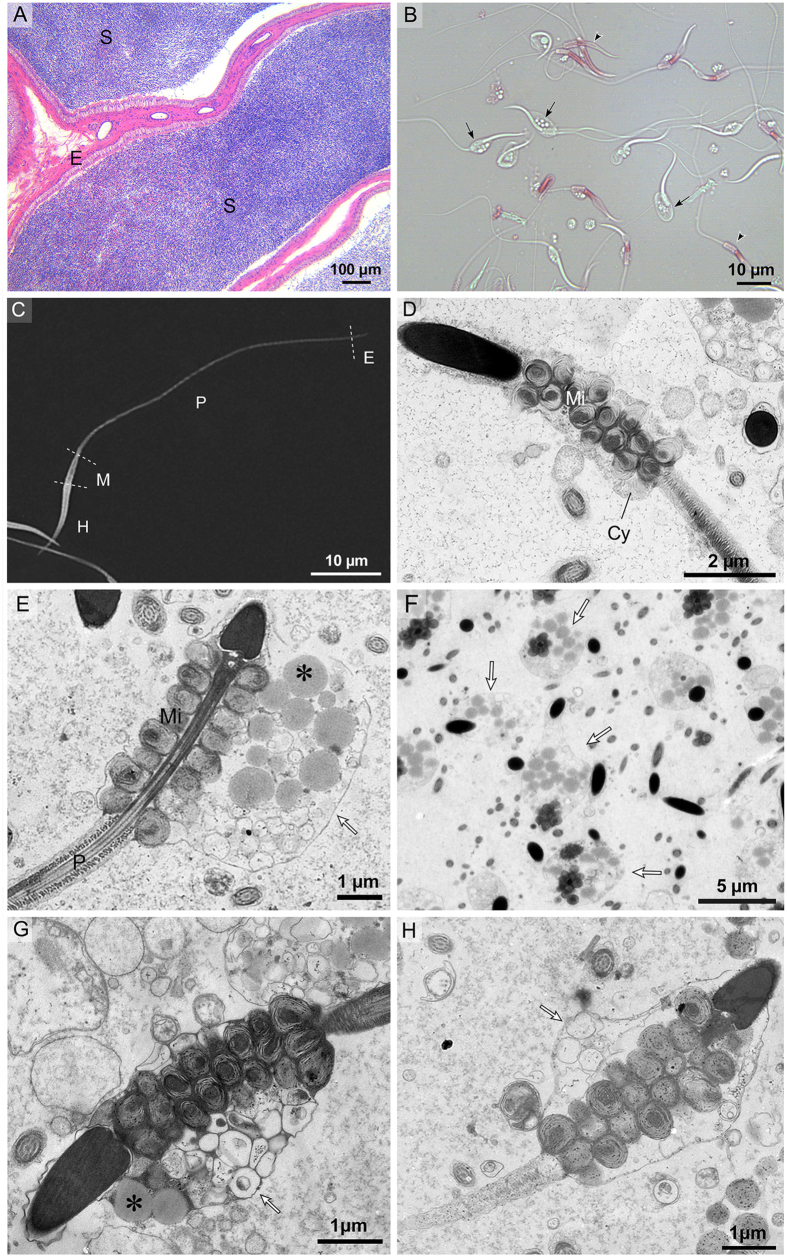
Spermatozoa in the epididymis of Chinese soft-shelled turtles. (**A**) numerous spermatozoa (S) stored in the epididymis (**E**), H&E stain. (**B**) the epididymal spermatozoa with large CD (↑) containing several large LDs and the mature spermatozoa (►) without CD, Eosin stain, phase contrast microscopy. (**C**) SEM of a mature spermatozoon composed of head (H), short midpiece (M), long principal piece (P) and short end-piece (**E**) in the later stage of sperm storage. (**D**) the wide midpiece (nearly double head width) with onion-like mitochondria (Mi) and little cytoplasm (Cy) in corpus epididymidis in the later storage stage, TEM. (**E**) longitudinal section of epididymal spermatozoon, note CD (↑ = 2.5 × 4 μm) attached along the entire midpiece and posterior head, which contained several large LD (*) and some membrane vacuoles (V), TEM. Mitochondria (Mi), Principal piece (P) in the early storage stage. (**F**) cross section of spermatozoon through CD (↑) in the early storage stage, TEM. (**G**) Spermatozoon with fewer LDs (*) within the CD (↑) in the late stage of sperm storage, TEM. (**H**) Spermatozoon only contained several membrane vacuoles within the CDs (↑) in the later storage stage, TEM.

**Figure 3:**
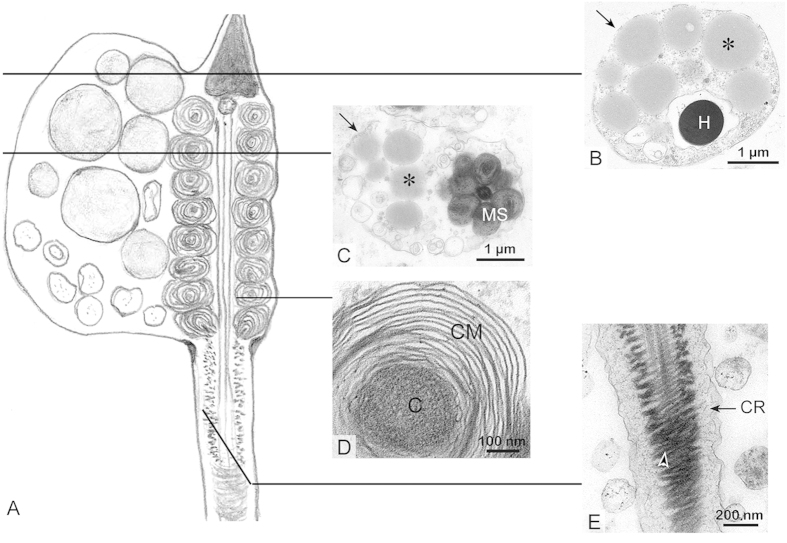
Spermatozoa stored in the epididymis. (**A**) the diagram of epididymal spermatozoon. Note the midpiece consisted of the CD with LDs and the onion-like mitochondria. The principal piece has a thick layer of cytoplasm around the fibrous sheath. (**B**) The posterior of spermatozoon head (H) with CD (↑) and LD (*) surrounding nucleus, TEM. (**C**) cross section through the midpiece displayed CD (↑) containing big LDs (*), located at a side of mitochondrial sheath (MS), TEM. (**D**) the onion-like mitochondrion with concentric laminated membrane (CM) enclosing a dense substrate core (**C**) in the midpiece, TEM. (**E**) the principal piece of the spermatozoon with cytoplasmic ring (CR) surrounding the developed fibrous sheath ( ⋩), TEM.

**Figure 4:**
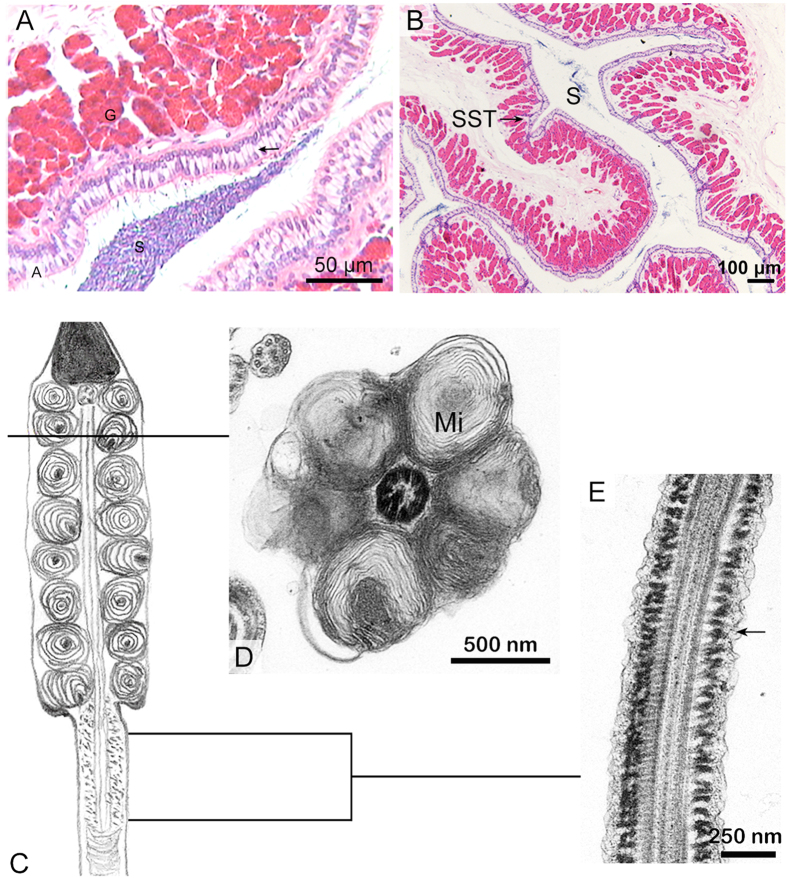
Spermatozoa in the early stage of sperm storage within the oviduct. (**A**) Spermatozoa stored within oviduct, (H,E) stain. Epithelium (↑), Spermatozoa (S), Gland (G). (**B**) The spermatozoa (S) stored in both the SSTs and lumen of the oviduct, H&E stain. (**C**) the diagram of spermatozoon showing no CD in the midpiece. (**D**) cross section through the midpiece maintained onion-like mitochondria (Mi) being close to each other, TEM. (**E**) the principal piece still possessing thin cytoplasmic ring (↑), TEM.

**Figure 5:**
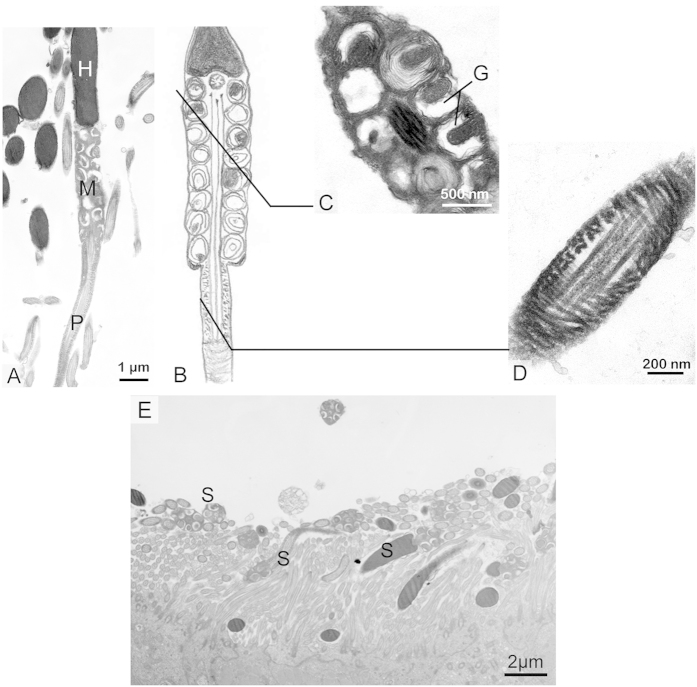
Spermatozoa in the intermediate stages of sperm storage within the oviduct. The schematic organization of spermatozoa (**B**) and corresponding cross section with TEM (**A,C,D**). (**A**) longitudinal section spermatozoon through head (H), thin midpiece (M) and principal piece (P), with the width ratio of spermatozoa head to mitochondrial sheath approximately one to one, TEM. (**B**) the diagram of stored spermatozoon. (**C**) diagonal plane section through the midpiece of the spermatozoon showing gaps (G) that appeared between the concentrated membrane layers of the mitochondria. (**D**) the cytoplasmic ring in the principal piece decreased markedly, TEM. (**E**) The structure variation of spermatozoa (S) in the SST was the same as that of the lumen, TEM.

**Figure 6:**
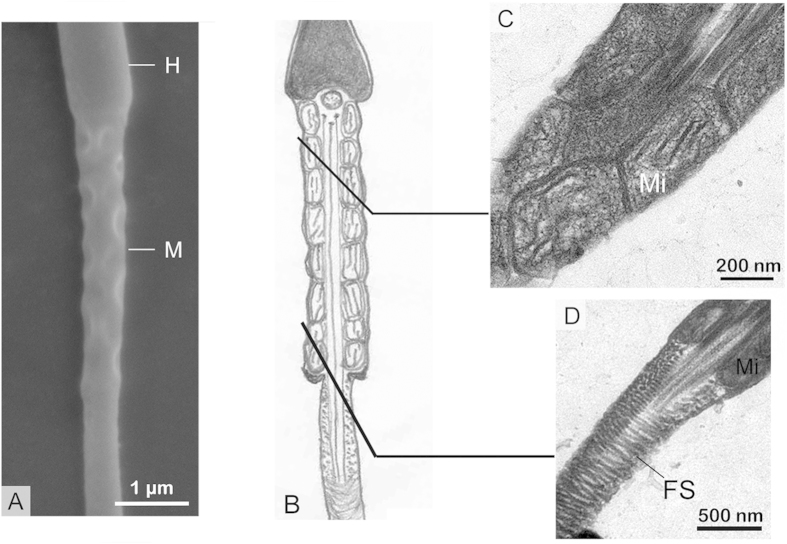
Spermatozoa in the later stages of sperm storage within the oviduct. (**A**) SEM image showing a slight spermatozoon with a thinner midpiece (M) compared with head (H). (**B**) the diagram of spermatozoon. (**C**) diagonal plane section through the midpiece of the sperm. Note that the mitochondria (Mi) returned to normal form, with a polygonal shape and tightly packed, TEM. (**D**) the principal piece almost without cytoplasm ring around fibrous sheath (FS), TEM.

Distinctive morphological features and transformations of the spermatozoa occurred during storage in both the epididymis and the oviduct (Fig. 1). Spermatozoa were found within the epididymis in the male (Figs 2 and 3) and within the oviduct in the female (Figs 4, 5 and 6) throughout the year. In oviduct, spermatozoa can be stored in both the lumen (Fig. 4A) and the SSTs (Fig. 4B). Based on the reproductive cycle of this turtle, sperm storage in the oviduct can be divided into three stages: early (summer and autumn), intermediate (winter) and late (spring). The mature spermatozoa had a long principal piece and a short midpiece (Fig. 2C), which was 61.28 ± 1.79 μm long (mean ± SE; n = 107) (Fig. 2C). Schematic representations of spermatozoa are shown in Fig. 1 and the following figures in each stage of sperm storage.

### Characteristics of the spermatozoa in the epididymis

The caput, corpus and cauda epididymides of the Chinese soft-shelled turtle contained large volumes of spermatozoa throughout the year (Fig. 2 and Supplementary Fig. S1 online). A large CD, 2.5 × 4 μm, was attached along the entire midpiece and posterior head of most of the epididymal spermatozoa (Fig. 2B,E,F and 3A–C). There were several lipid droplets (LDs) inside the CDs, and each of the LDs had a diameter of approximately 0.5 μm (Fig. 2B,F). From later autumn to later spring, corresponding to the early stage and later stage of sperm storage, the average number of LDs decreased from 7.01 ± 0.08 to 1.35 ± 0.07 (mean ± SE; counted fifty epididymal spermatozoa from each turtles), as representatively shown in Fig. 2E and Fig. 2G. The number of vacuoles inside the CDs increased from 4.14 ± 0.35 to 14.5 ± 0.41 (mean ± SE; n = 50). Some CDs just contained several vacuoles instead of LDs in later spring (Fig. 2H). The CD attachment site was retained along the midpiece and posterior head of the spermatozoon, which did not migrate down along the principle piece of the tail, from the caput to the cauda epididymidis (Fig. S1). Eight to fifteen concentric laminated membranes around a dense substrate core formed typical onion-like mitochondria in the midpiece (Fig. 3D), which had a diameter of 740.86 ± 77.52 nm (mean ± SE; n = 86). In the principal piece, a thick layer of cytoplasm surrounded the developing fibrous sheath (Fig. 3E).

### Modification of the spermatozoa in the oviduct

Many spermatozoa were found in the turtle oviduct (Fig. 4A). The structural variation of spermatozoa from the oviduct lumen was consistent with those from the SST (Fig. 5E). A CD was not attached to the midpiece, and the mitochondria maintained the onion-like ultrastructure and remained close to one another in the early stage of sperm storage (August and October) (Fig. 4C,D). At the beginning of the intermediate stages (January), gaps or lacunae appeared between the concentrated membrane layers of the mitochondria in the midpiece (Fig. 5A–C), and the volume of mitochondria was reduced, leading to a thin midpiece of almost the same width as the posterior end of the head (Fig. 5A). In some spermatozoa collected in later stages of sperm storage in May, the mitochondria returned to their normal structure of a double membrane with cristae (Fig. 6B,C). The mitochondria were polygonal and tightly packed, and the midpiece was thinner than the posterior end of the sperm head.

A cytoplasmic ring surrounded the fibrous sheath in the early stage of storage (Fig. 4E). However, the cytoplasmic ring of the principal piece became thinner in the intermediate stage of storage. Eventually, the cytoplasmic membrane tightly covered the sperm head and tail (Figs 5D and 6D).

## Discussion

The mechanisms for the storage of spermatozoa exhibit extraordinary diversity *in vivo*, but the mechanisms that maintain spermatozoa for a long time in the reproductive tracts of reptiles remain obscure. In the present study, we found that the turtle spermatozoa developed distinctive morphological features with noticeable differences between male and female turtles, which might have important roles for the survival of spermatozoa during long-term storage.

### CDs might assist epididymal sperm storage

Most of the cytoplasm of spermatids is removed and phagocytosed by Sertoli cells during sperm formation occurs in the testis^23^. However, a small portion of cytoplasm is generally retained as a CD on the flagella of spermatozoa during sperm transit through the epididymis in mammals^24^. The CD is not a useless residual cytoplasm on the spermatozoa, as it has been reported that CDs play a role in osmoadaptation by allowing water to enter or exit in the cell^25^. Moreover, several classes of enzymes have been identified in CDs, including lysosomal hydrolases, glycolytic enzymes and intermediate metabolic enzymes^26^,^27^,^28^,^29^,^30^. Yuan *et al.* have suggested that CDs serve as an energy source, providing the energy (i.e., ATPs) required for the continued maturation of epididymal spermatozoa^19^. In *Uraeotyphlus narayani* (Amphibia), the live spermatozoa possess a CD, which is shed from spermatozoa that have ceased motility^20^. Lipid droplets always provide energy to different cells^31^. In the present study, a much larger CD with several LDs was observed, and the number of LDs decreased after storage in the epididymis, which implied that CDs may be endogenous energy sources that allow sperm cells to use fatty acids as fuel during development and long-term storage in the epididymis. In most mammalian species, the droplet migrates down along the sperm midpiece during epididymal transit^32^. In this process, most of the cytoplasm, including some enzymes, is removed from the midpiece^33^. In this study we found that the CD was always retained adjacent to the midpiece and posterior head of the spermatozoon from the caput through the corpus to the cauda epididymidis. In painted turtle spermatozoa, the CDs were detached from the sperm midpiece in a coordinated manner shortly before the commencement of fall mating and are not observed on spermatozoa recovered from the oviduct of females^34^. The fact that CDs were retained on the spermatozoa in the epididymis but not in the oviduct suggested that the CDs play primary roles in epididymal sperm storage in the Chinese soft-shelled turtle.

### Mitochondrial modification of spermatozoa in the oviduct

The exact function of mitochondria in spermatozoa is controversial because they have extensive morphological heterogeneity. Long-term adaptations to various rates of ATP use can be achieved by modifying the number, morphology and location of mitochondria^35^,^36^. Mitochondria regulate different aspects of reproductive function, but these aspects are not uniform throughout the animal kingdom^37^. In insects, the mitochondria fuse to form giant mitochondrial derivatives^38^, which can drive the elongation of sperm^39^. In mammals, the spermatozoa show considerable metabolic flexibility, and many can function effectively on glycolysis without the need for mitochondrial energy production^40^. The onion-like mitochondria have been observed in certain reptiles, including *Chrysemys picta*^41^ and *Alligator mississippiensis*^42^. However, there have been very few studies of the function and fate of this type of mitochondria within the female reproductive tract. Our data showed that the mitochondria of spermatozoa in the epididymis and the early-stage oviduct were a typical onion-like shape with 8–15 layers of closely apposed membranes around a dense substrate centre. This configuration may not allow for the presence of ATP syntheses by the OXPHOS pathway as has been shown for steroid-producing cells^43^. In our study, when the spermatozoa were transferred into the turtle oviduct, the number of membrane layers decreased, and gaps gradually emerged between membrane layers in the mitochondria. Furthermore, mitochondria returned to the normal double membrane organelle at a later stage of storage, which implied a change in the mitochondrial metabolism in turtle spermatozoa, that may allow ATP production in the midpiece via the OXPHOS pathway as in the mitochondria of cells with a common morphology. Froman *et al.* proposed that spermatozoa stored in the hen oviduct are powered by oxidation of endogenous long-chain fatty acids, perhaps originating from the outer mitochondrial membrane^44^. The degradation/digestion of the mitochondrial lipoprotein membrane may provide an energy and/or nutrition during long-term sperm storage in the turtle spermatozoa. Alternately, the degradation/digestion inner membrane of mitochondria may cause cellular damage from reactive oxygen species. However, the inner membrane can recover a normal structure in the turtle spermatozoa, which may represent a special repair mechanism.

With respect to the mechanisms underlying the morphological changes of mitochondrial cristae, the presence of ATP synthase oligomers has been described as being essential to the maintenance of the mitochondrial cristae ultrastructure in yeast^45^. Indeed, destabilisation of the interactions between monomers has been shown to alter the organisation of the mitochondrial membrane, leading to the formation of onion-like structures similar to those observed in some mitochondrial pathologies^45^,^46^. These findings indicate that morphological changes of mitochondria can change the pathway of ATP production. Recent data suggest a physiological role for ATP synthase oligomerisation in the regulation of the atypical mitochondrial cristae shape observed in human syncytiotrophoblasts^46^. It is now widely accepted that there is a strong relationship between dimerisation/oligomerisation of ATP synthase and cristae morphology^47^,^48^,^49^. Nevertheless, the precise molecular and cellular mechanisms are not yet clear. Our previous work showed that normal mitochondria in early spermatids develop into onion-like mitochondria in later spermatids and immature spermatozoa when spermatogenesis takes place in the testis and epididymis of Chinese soft-shelled turtles^50^,^51^. In the present study, sperm mitochondria maintained the onion-like structure during sperm storage in the epididymis, but recovered the more common cristae configuration during later stages of storage in the oviduct. To our knowledge, this mitochondrial modification is first observed in vertebrate spermatozoa. Most mechanistic studies of the relationship between ATP synthase oligomerisation and mitochondrial morphology have been carried out with yeast cells^45^ because fewer vertebrate cells are available for study. Thus, the turtle spermatozoon, which has typical onion-like mitochondria, could be a useful cell model for mitochondrial research.

### Dense fibrous sheath support spermatozoa stored in the genital tract

Recent evidence suggests that glycolysis, which takes place along the fibrous sheath in the principal piece, may provide most of the energy to sustain sperm motility^52^,^53^,^54^, although mitochondria in the midpiece are traditionally considered the source of energy for sperm movement in mammals. It has been demonstrated that the fibrous sheath functions as a scaffold for localized proteins involved in signalling cascades^21^,^22^, which are critical for normal flagellar function^55^,^56^. Indeed, glycolytic enzymes seem to be compartmentalised in the fibrous sheath, a cytoskeletal element of the principal piece^57^,^58^, and ATPs can be produced in the principal piece through the glycolytic pathway. This is particularly true in species with long sperm tails because it is doubtful that sufficient ATPs could diffuse to the distal end of the flagellum^59^. In the present study, the spermatozoa of the Chinese soft-shelled turtle had a long flagellum with a developed fibrous sheath, which were the morphological conditions for ATP production in the principal piece through the glycolytic pathway. The sperm survive for a long time under anoxic conditions in the relatively impermeable environment of the oviduct, primarily because of the developed fibrous sheath in turtle spermatozoa, which provide more ATP with the glycolytic pathway.

Regarding the sperm’s energy supply, ATP production might vary to match energy demands in cells, and the balance between the glycolytic and OXPHOS pathways is known to vary between species^37^. During long-term sperm storage in the turtle, the spermatozoa underwent distinct morphological changes in energy-related organelles, probably so that they could utilize diverse pathways of energy metabolism to nourish and support spermatozoa at different stages of storage. The possible pathways were as follows: the glycolytic pathway and lipolysis in CDs were dominant in the midpiece of epididymal spermatozoa; the OXPHOS pathway in mitochondria with normal cristae in the midpiece was dominant in the oviduct at the later stages of sperm storage; and the glycolytic pathway was used in the fibrous sheath of the principal piece in both epididymal and oviduct spermatozoa. Our results suggest that the variation in sperm morphology implies a male strategy to produce alterable spermatozoa from long-living spermatozoa to maximize their fertilization success in different reproductive environments.

## Conclusion

This is the first report to address the cytological mechanism of long-term storage of spermatozoa in terms of the spermatozoa. Ultrastructural characteristics and their temporal transformations at different stages in the genital tracts were found for the spermatozoa, including large CDs with LDs, onion-like mitochondria, and developed fibrous sheath, all of which may be factors in the long-term storage of spermatozoa in the turtle. The spermatozoa have important roles for survival during long-term storage, in addition to supports received from the reproductive tracts.

## Methods

### Animals

All procedures with the turtle were conducted according to the Animal Research Institute Committee guidelines of Nanjing Agriculture University, China. Twenty male and twenty female Chinese soft-shelled turtles, sexually mature and aged 3–4 years, from a single pond in Nanjing, southeastern China were used in this study. Animals were rendered comatose using intraperitoneal administration of sodium pentobarbital (20 mg/kg) and killed by cervical dislocation. Animals were collected in August (summer), October (autumn), January (winter), and May (spring), 5 male and 5 female turtles at each time. Samples of the caput, corpus and cauda epididymides, oviduct and spermatozoa were collected immediately after death and fixed for light and electron microscopy respectively. The sampling procedures were approved by the Nanjing Agricultural University Veterinary College. The protocol was approved by the Science and Technology Agency of Jiangsu Province. The approval ID is SYXK (SU) 2010-0005. All efforts were made to minimize animal’s suffering.

### Light microscopy

The tissues were embedded in paraffin wax after fixation with 10% neutral buffered formalin for 24 h, and then serially sectioned (at 5 μm). The sections were stained with hematoxylin and eosin (Harry’s hematoxylin for 2 min and 1% eosin 30 s) and observed under an Olympus microscope (BX53; Olympus, Tokyo, Japan), camera (DP73, Olympus, Japan). The spermatozoa were collected and immediately stained with eosin 5 g/L (PBS, pH 7.4, 0.1 M) and examined under a phase contrast microscope (Olympus BX53).

### Transmission electron microscopy (TEM)

The samples were cut into small blocks, and fixed in a mixture of 2.5% (v/v) glutaraldehyde in phosphate buffered saline (PBS; 4 °C, pH 7.4, 0.1 M) for 24 h. The blocks were then rinsed in the same PBS, post-fixed for 1 h at room temperature in similarly buffered 1% osmium tetroxide, and washed in the buffer. The samples were dehydrated in ascending concentrations of ethanol, infiltrated with a propylene oxide–Araldite mixture, and embedded in Araldite. The blocks were sectioned, and the ultrathin sections (50 nm) were mounted on Formvar-coated grids, stained with uranyl acetate and lead citrate for 20 min per step. The sections were examined and photographed with a transmission electron microscope (TEM; Hitachi H-7650, Japan).

### Scanning electron microscopy (SEM)

Spermatozoa collected from the epididymides or oviduct were concentrated by centrifugation and resuspended in 2.5% glutaraldehyde and fixed for 3 h. After three washes in 0.1 M cacodylate buffer the samples were post-fixed in 1% osmium tetroxide for 1 h. After dehydration through a graded ethanol series, a drop of spermatozoa suspended in ethanol was placed on a glass cover slip, and the spermatozoa were allowed to settle. The samples were taken through critical point drying in CO_2_ and coated with gold^60^. Images were taken with a scanning electron microscope (SEM; Hitachi S-520, Japan). The morphometric parameters were measured from at least 30 spermatozoa.

## Additional Information

**How to cite this article**: Zhang, L. *et al.* Modification of sperm morphology during long-term sperm storage in the reproductive tract of the Chinese soft-shelled turtle, *Pelodiscus sinensis*. *Sci. Rep.* **5**, 16096; doi: 10.1038/srep16096 (2015).

## Supplementary Material

Supplementary Figure S1
