# Supplementary material for: Modification of sperm morphology during long-term sperm storage in the reproductive tract of the Chinese soft-shelled turtle, Pelodiscus sinensis
Source: Sci Rep. 2015 Nov 5;5:16096. doi: 10.1038/srep16096 (PMC4633597; doi:10.1038/srep16096)
Supplement: Supplementary Figure S1 [file srep16096-s1.pdf]

Modification of sperm morphology during long-term sperm storage in the reproductive tract of the Chinese soft-shelled turtle, *Pelodiscus sinensis*

Linli Zhang, Ping Yang, Xunguang Bian, Qian Zhang, Shakeeb Ullah, Yasir Waqas, Xiaowu Chen, Yi Liu, Wei Chen, Yuan Le, Bing Chen, Shuai Wang and Qiusheng Chen*

Laboratory of Animal Cell Biology and Embryology, College of Veterinary Medicine, Nanjing Agricultural University, Nanjing, Jiangsu 210095, PR China.

Correspondence and requests for materials should be addressed to Q.C (chenqsh305@njau.edu.cn)

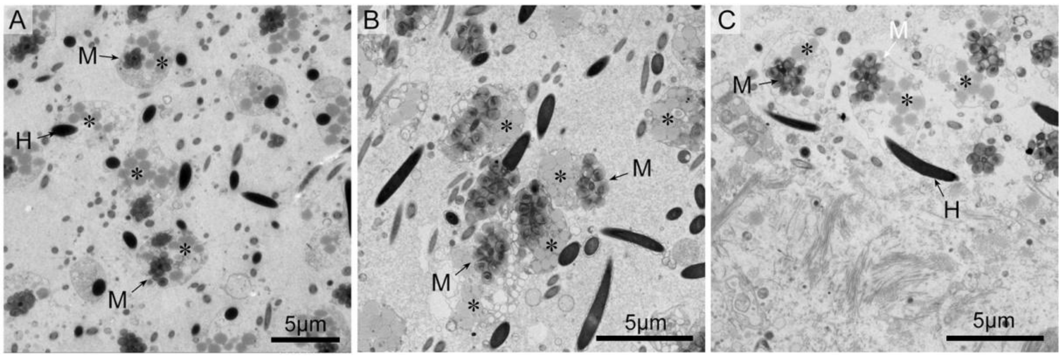

Supplementary Figure S1 | Spermatozoa in the caput, corpus and cauda epididymidis of Chinese soft-shelled turtles, TEM. (**A**) caput epididymidis, (**B**) corpus epididymidis, (**C**) cauda epididymidis. The cytoplasmic droplet was attached along the entire midpiece (M) and posterior head (H), from caput to cauda epididymidis. The large cytoplasmic droplet contained several large lipid droplets (*) and some membrane vacuoles.
